# Risk of Introduction of Classical Swine Fever Into the State of Mato Grosso, Brazil

**DOI:** 10.3389/fvets.2021.647838

**Published:** 2021-07-01

**Authors:** Daniella N. Schettino, Fedor I. Korennoy, Andres M. Perez

**Affiliations:** ^1^Department of Veterinary Population Medicine, Center for Animal Health and Food Safety, College of Veterinary Medicine, University of Minnesota, St. Paul, MN, United States; ^2^Animal Health Coordination, Instituto de Defesa Agropecuária de Mato Grosso (INDEA-MT), Mato Grosso, Cuiabá, Brazil; ^3^FGBI Federal Centre for Animal Health (FGBI ARRIAH), Vladimir, Russia

**Keywords:** classical swine fever, risk assessment, domestic pigs, wild boars, Mato Grosso, Brazil

## Abstract

Classical swine fever (CSF) is considered one of the most important diseases of swine because of the far-reaching economic impact the disease causes to affected countries and regions. The state of Mato Grosso (MT) is part of Brazil's CSF-free zone. CSF status is uncertain in some of MT's neighboring States and countries, which has resulted in the perception that MT is at high risk for the disease. However, the risk for CSF introduction into MT has not been previously assessed. Here, we estimated that the risk for CSF introduction into the MT is highly heterogeneous. The risk associated with shipment of commercial pigs was concentrated in specific municipalities with intense commercial pig production, whereas the risk associated with movement of wild boars was clustered in certain municipalities located close to the state's borders, mostly in northern and southwestern MT. Considering the two pathways of possible introduction assessed here, these results demonstrate the importance of using alternative strategies for surveillance that target different routes and account for different likelihoods of introduction. These results will help to design, implement, and monitor surveillance activities for sustaining the CSF-free status of MT at times when Brazil plans to expand the recognition of disease-free status for other regions in the country.

## Introduction

Classical swine fever (CSF), also referred to as hog cholera, is arguably one of the most important viral diseases affecting domestic and wild swine, and for that reason, the disease is notifiable to the World Animal Health Organization (OIE). The CSF's impact on the swine industry is associated with the mortality and reduction of productivity caused by the disease, and, most importantly, with disease-related trade restrictions, which results in important economic and social consequences for infected areas (1–3). CSF is caused by an enveloped RNA virus of the genus *Pestivirus* of the family Flaviviridae referred to as CSF virus (CSFv). The most common routes for CSF spread include oronasal transmission through direct or indirect contact with infected pigs, consumption of pig meat infected with the virus, and vertical transmission from an infected sow to her offspring (4–6).

Sixteen (15 states and 1 federal district) of the 27 administrative units of Brazil have been recognized by the OIE as CSF-free since May 2016; those 16 administrative units constitute the majority of the country's national pig production. The state of Mato Grosso (MT) is the fifth largest pig producer in the country, with 2,590,872 heads corresponding to approximately 8.7% of the Brazilian pig herd and is located in the CSF-free zone of Brazil. Most (*n* = 1,933,248 pigs, 74.6%) of MT's pig population is concentrated at 1.3% (*n* = 550) of the premises registered as commercial pig farms in the state, whereas the remaining 657,624 (25.4%) pigs are located in 43,398 backyard (subsistence) farms (7). There are also seven multiplier farms in MT, and commercial operations are divided into farrow-to-finish, sow, and finishing farms. Commercial pig farms are highly concentrated in municipalities at the central-northern region of the state. Although CSF has never been reported in MT, the state is adjacent to the non-CSF-free zone of Brazil in the north (states of Amazonas and Para) and Bolivia (where the CSF status is uncertain) in the southwest. For that reason, there is a perception among MT swine producers that the state is at high risk for the introduction of CSFv. Additionally, the last CSF outbreaks reported in Brazil (2009 and 2018/2019/2020) affected backyard pig farms in the non-CSF-free zone (8) increasing concerns among swine farmers in the CSF-free area.

Free roaming of CSF-infected wild boars, which are considered an exotic and intruder species in the Brazilian territory (9), may result in the introduction of CSF into MT. Additionally, although the movement of pigs and pork products is only allowed between states in the CSF-free zone, the CSF-free zone is quite extensive and includes a number of Brazilian States. For that reason, if an outbreak occurs in a state other than MT, there are chances that infected pigs may be moved into MT prior to the time of outbreak detection, when animal movements would be banned.

Risk assessment is an epidemiological tool frequently used by countries to assess the risk for transboundary animal diseases (TADs) such as CSF, African swine fever (ASF), and foot-and-mouth disease (FMD). Many studies have been developed to assess the risk of introduction of these diseases into free areas, mostly through movement of live animals or animal products as part of international trade, which is one of the reasons for performing risk assessments according to the OIE. In the early 2000s, most published risk assessments were related to FMD and CSF and considered pathways such as pig movement, pig products (semen, pork), and fomites (2, 10–12). After the incursion of ASF into Georgia in 2007, many risk assessments were performed for ASF introduction into free areas, and wild boars started to be included as potential pathways (13–16). Risk assessments are most frequently performed at the national level to propose risk mitigation actions associated with international contacts, but for countries in which regulations are implemented by states rather than federal governments, such as Brazil, there are also benefits in estimating the risk at the subnational level (12).

The objective of the study here was to rank MT municipalities in terms of their risk for CSFv introduction, either through wild boar movements or through legal movement of commercial pigs, and to compare those ranks to evaluate the correlation at the municipality-level risk of entry through those two pathways. The results will help to inform the design of surveillance strategies and allocation of resources in MT with the ultimate objective of preventing or early detection of a hypothetical introduction of CSFv into the State.

## Materials and Methods

### General Approach

The risk for CSFv introduction into MT through two alternative pathways, namely, (i) the movement of live pigs assuming a hypothetical CSF outbreak in the CSF-free zone of Brazil and (ii) free ranging of wild boars, described in the following sections, was assessed at the state and municipality levels in MT. Municipalities were subsequently ranked in terms of the risk associated with each pathway, and ranks were compared to evaluate the correlation between pathways.

### CSFv Introduction Through Movement of Live Pigs—Assessing Risk for Commercial Farms

#### Animal Data Sources

Official data from MT's Official Veterinary Service (INDEA/MT) regarding the legal movement of pigs into MT from 2016 through 2018 were used (7). All shipments originated from CSF-free states, given that pig movements from non-CSF-free states are banned, were retrieved from the INDEA/MT database. Movements for slaughtering and/or fair purposes were screened out because slaughter is a dead end for disease transmission, and pig fairs are rare in MT. Subsequently, only between-farm movements were considered for the analysis.

#### Analytical Framework

A stochastic risk assessment model was fitted to estimate the probability of introduction of CSFv into MT *via* movement of live pigs during a 1-year time period, which was assessed both at the state and municipality levels. For the estimate of risk at the state level, we considered the total number of pigs that were shipped to MT from the states that are part of the CSF-free zone and, hence, allowed to trade with MT, given the hypothetical scenario of one undetected epidemic on the CSF-free zone of Brazil. For the probability of introducing the disease into any municipality of MT, we considered the number of animals that were shipped into each municipality of MT. The annual risk for CSF introduction into MT farms through pig movements (Rpm) was quantified assuming a binomial model of the form

$$Rpm=1-{\left(1-Psm\;\right)}^{Nsm}$$

where N*sm* is the number of pigs shipped from the CSF-free zone into each municipality *m* of MT before detection of the outbreak in the free zone; for the estimates at the state level, the total number of pigs shipped into MT was used. P*sm* was the probability of introduction of one infected animal. The value of P*sm* was the same for each municipality *m* and for the state of MT, and it was computed as the product of four conditional probabilities (P1–P4) describing the nodes of the risk pathway, which were modeled in a scenario tree (10, 13, 17). Nodes were parameterized (Table 1) following principles explained in detail elsewhere for selecting distributions (21), and the approach was similar to risk assessments done for the introduction of CSF (11) and FMD (2) in Spain, and ASF (13) in the European Union.

**Table 1:** Parameterization of a quantitative assessment of the risk of introduction of classical swine fever (CSF) into the state of Mato Grosso (MT), *via* legal movement of pigs and assuming a CSF outbreak in the disease free-zone of Brazil.

**Input**	**Parameter**	**Distribution**	**Value**	**Source of information**
Population of commercial pigs in free zone (NT)	NT	Normal	μ^*^: 22,758,504 σ^**^: 1,529,008.972	Database MAPA-BR (18)
Total number of commercial farms—herd number (NH)	NH	Normal	μ^*^: 25,902 σ^**^: 784.621	Database MAPA-BR (18)
Average herd size (H)	H	Equation	NT/NH	Model equation
Intraherd prevalence (IP)	IP	Pert	Min: 0.05 Most likely: 0.4 Max: 1	Martínez-López et al. (11)
Expected undetected outbreaks (EO)	EO	Pert	Min:1 Most likely: 6 Max: 39	Martínez-López et al. (11)
Number of pigs in free zone expected to be infected before the detection of the outbreak (NI)	NI	Equation	IP * H * EO	Model equation
Probability of importing an infected commercial pig from free zone (assuming an outbreak before detection) (P1)	P1	Beta	α1 = NI + 1 and α2 = NT – (NI + 1)	Adapted from Martínez-López et al. (11); Database MAPA-BR (18)
Probability of infected pig surviving the infection (P2)	P2	Pert	Min: 0.63 Most likely: 0.78 Max: 0.932	Martínez-López et al. (11)
Probability of infected pig surviving shipment (P3)	P3	Pert	Min: 0.908 Most likely: 0.9973 Max: 0.9995	Murray and Johnson (19)
Probability of quarantine in destination (Pq)	Pq	Beta	α1 = 130.71 and α2 = 15.41	Martínez-López et al. (2); Martínez-López et al. (11)
Probability of detection during quarantine (Pd)	Pd	Beta	α1 = 1.33 and α2 = 34.16	Martínez-López et al. (11); Mur et al. (13)
Probability of non-detection of infected animal at destination and of animal establishing contact with susceptible in MT farm (P4)	P4	Equation	1 – Pq * Pd	Martínez-López et al. (11); Mur et al. (13)
Time of detection in days (Td)	Td	Pert	Min: 11 Most likely: 40 Max: 127	Bronsvoort et al. (10), Pineda et al. (20), and OIE -WAHIS (8)
Number of pigs shipped to MT (and to each municipality m)	*n*	Poisson-lognormal	μ^*^ and σ^**^ of number of pigs sent from states s to MT [and each municipality of destination *m* (2016–2018)]	INDEA/MT database (7)

* *Mean*,

** *standard deviation*.

The first node (P1) of the scenario tree (22) was the probability of importing an infected commercial pig from the CSF-free zone during the silent phase of the epidemic, i.e., before detection of the Official Veterinary Service (OVS) in the origin (the CSF-free zone of Brazil). A beta distribution was used to calculate this probability, of the form α1 = NI + 1 and α2 = NT – (NI + 1), where NI is the “*Number of pigs, expected to be infected in the free-zone before the detection of the outbreak*,” and NT is the “*Population of commercial pigs in the CSF-free zone (NT).”* The calculation of these parameters is described later in this section.

The second and third nodes, denoted as P2 and P3, were the probabilities that the infected pig survived infection and shipment, respectively, for which we used a Pert distribution parameterized with data extracted from the literature (2, 11).

The last node of the scenario tree (P4) represented the probability that an infected imported pig established contact with a susceptible pig in a farm in MT, causing a CSF outbreak, i.e., assuming a failure of quarantine and detection by OVS at the municipality of destination *m*. This probability was calculated as 1 – Pq * Pd, where Pq is the probability of quarantining the animal at the destination, and Pd is the probability of detecting the disease during that quarantine (11, 23).

For the calculation of the “*population of commercial pigs in free zone (NT)”* variable, we used a normal distribution (normal μ, σ), considering as mean (μ) the total number of pigs in commercial pig farms at the CSF-free zone in 2017, except MT, and σ is the standard deviation of the total number of commercial pigs at the CSF-free zone during the period 2014–2017. This input was one of the components used to calculate P1. Data required to estimate the parameter NT was obtained from the Brazilian Ministry of Agriculture, Livestock and Food Supply (MAPA/BR) (18).

Values for the “*total number of commercial farms - herd number (NH)*” variable were calculated using a normal distribution (normal μ, σ), considering as mean (μ) the total number of commercial pig farms at the CSF-free zone in 2017, and σ is the standard deviation of the total number of commercial pig farms in the period 2014–2017. This input was used to calculate the average herd size (H). Data required to estimate the parameter NH were obtained from the Brazilian Ministry of Agriculture, Livestock and Food Supply (MAPA/BR) (18).

The variable “*Number of pigs, expected to be infected in the free-zone before the detection of the outbreak (NI)”* was calculated by the equation IP * H * EO, where intraherd prevalence (IP), the average herd size in the CSF-free zone (H), and the number of expected undetected outbreaks at the origin (EO) were multiplied to generate the number of pigs expected to be infected at the CSF-free zone during an outbreak in the silent phase (NI), that is, before the detection of the index case of CSF by the OVS in the origin (11). The parameters *average herd size (H), intraherd prevalence (IP), and expected undetected outbreaks (EO)* were calculated as explained in the following paragraphs.

Because states in the CSF-free zone have the same sanitary status regarding CSF, and they are allowed to trade pigs between them, we assumed the CSF-free zone as one single unit, whereas the risk for introduction of CSF was stratified for each municipality of destination *m*. The *average herd size (H)* was approximated as the *NT/NH* ratio in the CSF-free zone.

The “*intra-herd prevalence (IP)*” was calculated using a Pert distribution; although the incubation period of CSF is generally 4–10 days, under field conditions, CSF is expected to show unspecific symptoms at the beginning of an outbreak, which can delay the detection of infected herds in 2–4 weeks, increasing the intraherd prevalence at the moment of detection by the OVS (24, 25).

The “*expected undetected outbreaks (EO)*” is defined as the number of herds that would be infected by the time when a hypothetical epidemic in the CSF-free zone was detected and pig movements into MT banned. EO was assumed to follow a Pert distribution with a minimum of one undetected outbreak (the index case), and the most likely and maximum equal to the number of herds that were affected before the detection of the CSF epidemics in Spain in 2001, and in The Netherlands in 1997, respectively (11).

To adjust the number of pigs that would be sent to MT between the beginning and detection of the outbreak in the CSF-free zone, we estimated the *time-to-detection (Td)*, i.e., the length in days before the epidemic is detected, and movements into MT are banned. Under field conditions, the detection is expected to take longer than the incubation period. A Pert distribution was used for modeling *Td*, with the minimum, most likely, and maximum values being those reported in Colombia, in Ceará (a state of Brazil in the CSF non-free zone in which outbreaks occurred in 2018), and the recommendation of the European Union on the parameter that should be used when there is no information available, respectively (8, 10, 20).

The number of pigs that were shipped from the CSF-free zone was estimated considering the number of pigs that came from states *s* into MT and into each municipality *m* of MT during the years 2016–2018. For each municipality of destination *m* during the period of the study, we grouped the movement from 2016 to 2018 and computed the mean (μ) and standard deviation (σ) of the total number of pigs that were sent to each municipality *m* from states of origin *s*, during this period (7). Then, the number of pigs annually shipped into MT and into each municipality m (n) from the CSF-free zone was assumed to follow Poisson-lognormal distributions, with mean (μ) and standard deviation (σ) estimated for MT and for each municipality *m*, respectively. The number of pigs that each municipality *m* of MT received is listed in the Supplementary Material. The number of pigs that would be shipped from the CSF-free zone before detection of the outbreak in the free zone (N*sm*) was subsequently computed for MT and for each municipality *m* as the number of pigs shipped per day (n/365) multiplied by the time-of-detection (in days) of an outbreak in the CSF-free zone (Td), so that:

$$Nsm={\left(n/365\right)}^{*}\;Td.$$

A spider graph was generated in Excel to evaluate what parameters (Table 1) mostly contributed to changes in the mean risk for the introduction of CSF into MT, i.e., assessing the sensitivity of the model to the uncertainty and variability associated with its parameterization. For that sensitivity analysis, we selected the first (Q1), second (median—Q2), and third quartile (Q3) for the distribution of each parameters evaluated, i.e., P1, P2, P3, P4, Td, and *n*. The median was the measure of central tendency, and Q1 and Q3 were measures of dispersion. We systematically calculated the final risk probability with different situations for each parameter at a time when the others were kept fixed in the median (second quartile) as the central tendency.

#### Computational Environment and Software

The stochastic model was implemented in the @Risk 8.0 software (26) and run through 10,000 iterations. Results were spatially visualized using Arc GIS version 10.5.1 (27).

### CSFv Introduction Into MT Through Wild Boars—Assessing Risk for Backyard Farms

#### Animal Data Sources

Pig farms registered in the INDEA/MT database by July 2019 were retrieved, including data on type of farms (subsistence, commercial), their geographic location, and the total number of pigs per farm (7).

Additionally, data regarding active surveillance activities for CSF in MT pig farms from 2016 to 2018 were retrieved to determine the presence or absence of free-range wild boars at those premises. Records of visits were organized in a dataset, and records repeated on any given farm were removed manually. Presence of wild boars was reported in 1,688 (24.7%) of the 6,827 visited farms (7). Data were used to estimate the distribution of wild boars fitting a maximum entropy (MaxEnt) model and procedures described elsewhere (28). Briefly, farms in which wild boars were reported were geolocated. Then, data on 27 environmental layer variables assumed to influence the presence of wild boar population in MT were retrieved, including 19 rasters from the WorldClim online database for the period 1970–2000 at a spatial resolution of 5 arc-min (~10 km). These variables (WorldClim) are derived from records of temperature and precipitation. Consequently, it is possible for at least some of those 19 variables to be highly correlated with each other, potentially leading to issues with colinearity; for those reasons, there is a need to remove highly correlated variables from the final model (29). The human influence or anthropogenic impact was approximated using the human footprint raster obtained from the Socioeconomic Data and Applications Center from Wildlife Conservation (WCS) and Center for International Earth Science Information Network (CIESIN)—Columbia University. The global footprint raster is a global dataset of 1-km grid cells, created from nine global data layers covering human population pressure (population density), human land use and infrastructure, and human access (30). The variable altitude/elevation data (referred to as SRTM) were extracted using DIVA-GIS, which is a free computer program for mapping and geographic data analysis with ready-to-use downloading raster. SRTM stands for Shuttle Radar Topography Mission (SRTM), and it is a 3 arc, i.e., 30 s of resolution, raster created with data from the National AeroSpatial Agency (NASA) representing a near-global set of land elevations (31). For the variable land cover, vegetation, crops, temperature, and isothermality, raster data were extracted from IPUMS Terra—Integrated Population and Environmental data, which is a global-scale framework data that allowed extraction data by country level (Brazil) (32). The vegetation index was extracted as a product of MODIS Land Cover, which is produced by NASA, and from this was selected the specific vegetation index for MT with a 250-m resolution (33). The global total irradiation was acquired by downloading a raster data from Global Solar Atlas, which is published by the World Bank Group, and prepared by Solargis, with a resolution of 250 m (34). Our choice of final variables was ultimately determined by the procedure of reducing multicolinearity but keeping variables that make sense for the purpose of detecting the wild boar population distribution in MT. Thus, a colinearity diagnostic was performed to screen out highly correlated environmental variables. The redundant variables were identified by the Raster package in R studio (35) and removed from the model if the meaning of the variable would not hamper the final model. Subsequently, only 15 environmental variables were used in the model (Table 2). The prediction value generated by each geographic coordinate was summed by each polygon, which were the 141 municipalities of MT. Then, these set of values were separated by the median, and the values were set as 50% high and 50% low density. This final information regarding the high/low density for wild boar population per each municipality of MT was included in the model generated for the risk calculation of introduction of CSF in MT *via* wild boars and explained in detail in the following section Analytical Framework.

**Table 2:** Environmental variables used to predict the distribution of wild boars in the state of MT, using a maximum entropy (MaxEnt) model.

**Type**	**Variable name**	**Description**
Human influence	hfp	Human footprint. Represents the impact of humans in the environment
Climate	bio 3	Isothermality (BIO2/BIO7) (×100)
	bio 7	Temperature annual range (BIO5–BIO6)
	bio 8	Mean temperature of wettest quarter
	bio 13	Precipitation of wettest month
	bio 15	Precipitation seasonality (coefficient of variation)
	bio 18	Precipitation of warmest quarter
	bio 19	Precipitation of coldest quarter
	isotherm	Oscillations of day–night temperature comparing summer/winter
Altitude/elevation	bralt	Shuttle Radar Topography Mission (SRTM) with 3 arc seconds (30 s) of resolution
Vegetation	crop	Area used as a cropland
	landcover	Global land cover area reference
	veg	Cropland/natural vegetation mosaic
Vegetation index	sdat	The vegetation index variation from the years 2000–2001 and 2003–2004, specific for Mato Grosso
Solar incidence	gti	Global total irradiation

#### Analytical Framework

The assumption here was that wild boars in Bolivia and in Brazilian states outside of the CSF-free zone may carry the CSFv and pass freely through the MT borders. We also assumed that the risk at the municipality level would be influenced by the domestic pig density, wild boar density, backyard farming share, shared border with CSF-infected zone or Bolivia, road density, and human population density in the state. The values of those variables were dichotomized (high/low or yes/no). Specifically, (a) pig density was calculated as the number of pigs in each municipality divided by the area in km^2^ and dichotomized using the median value (50% high, 50% low). The number of commercial pigs in the municipality was included in the computation, in addition to backyard pigs, to account for the probability of contact between backyard pigs and commercial pigs, and because backyard farming was specifically included as a separate variable, thus, accounting for that factors in the computations (36). (b) Backyard farming share was calculated as the number of backyard farms divided by the number of farms per each municipality and dichotomized using the median value (50% high, 50% low); this risk factor can play an important role in the dynamic of CSF due to low biosecurity and little interaction with veterinary services (37). Values for calculation of (a) and (b) were extracted from the database of the MT OVS (7). (c) Human density was calculated as the population estimated in the last national census conducted in 2010 (38) for each MT municipality, divided by the area (km^2^) of each correspondent municipality of MT, and dichotomized using 5 habitants/km^2^ as the threshold (high, low); the 5 habitants/km threshold was set up because it was the approximate midpoint between the median (2.29 habitants/km^2^) and mean (6.76 habitants/km^2^) densities and that resulted on an acceptable 1:3 ratio for the classification of districts as high or low density—alternatively, the use of the mean and median as cutoff values for the classification did not affect the results of the regression model (data non shown). Human density was included as a proxy for the movement of people (tourists or workers) that can carry contaminated food that can be disposed and accessed by wild boars (20, 39–41). (d) Road density was calculated using ArcGIS, considering the layers of municipalities and layers of roads of MT and dichotomized using the median (50% high, 50% low); road density was included because the introduction and spread of the disease may be influenced by human activities that could increase the risk for contacts with wild boars (37). (e) Wild boar density was estimated aggregating the results of the maximum entropy (MaxEnt) (described in the section Animal Data Sources for this pathway) prediction model at the municipality level and dichotomized using the median (50% high, 50% low). Wild boar density is important not only because of the susceptibility of wild boar to CSF infection but also because if infected, populations of wild pigs may be the primary source for CSF introduction in domestic pig herds (40). Dichotomization of the variable was required to incorporate it in the regression model and also to increase the accuracy of the MaxEnt predictions. (f) Shared border with a non-CSF-free state or country was estimated using ArcGIS and dichotomized (yes, no). The relative contribution of each variable to the final risk was assumed to be similar to the weight estimated by a panel of experts for the risk of introduction of African swine fever (ASF) into a free region from a neighboring infected country described in detail elsewhere (41). Briefly, the model approach was based on a factorial design to identify 10 representative scenarios of the combination of parameters hypothesized to influence the risk of introduction of ASF (domestic pig density, wild boar density, backyard farming share, share border to a country that is infected with ASF, road density, and human density). Each scenario was referred to as hypothetical Region A to hypothetical Region J (*n* = 10) representing different epidemiological conditions. International experts, which were chosen by snowball sampling technique after consultation with the OIE reference laboratories for ASF in Spain, the UK, and the National Reference Laboratory of the Russian Federation, were requested to rank the 10 hypothetical scenarios in terms of their likelihood of serving as a port of entry for ASF into the country, where 1 meant the lowest risk, and 10 meant the highest risk for introduction of the disease in districts of a free country, and the hypothetical scenarios were categorized by a combination of dichotomized (high/low, yes/no) risk factors listed before. An ordinal logistic regression model was fitted to estimate the relative weight that the experts implicitly gave to each of the variables (pig density, backyard farming share, human density, road density, wild boar density, and share border with a non-CSF-free region), as approximated by the value of the regression coefficients. A risk score of the introduction of CSF through wild boar (Rbm) was subsequently computed assuming an increase by factors of Rbm = β_0_ + 3.39 * pig density + 4.16 * backyard farming share + 0.55 * human density + 0.67 * road density + 3.4 * wild boar density + 2.34 * share border with non-CSF-free region for municipalities categorized as high (or yes), compared with those categorized as low (or no). Rbm was computed for each of the 141 municipalities in MT as the sum of the dichotomized values of the risk predictors weighted by an increase in risk assumed for each of the factors. Finally, municipalities were ranked in terms of the computed value of Rbm.

#### Computational Environment and Software

The MaxEnt software (42) was used for computing the maximum entropy model of wild boar distribution. The correlation between environmental layers was conducted in RStudio Team (2019) version 3.5.3 (35) using “raster” and “rgdal” packages; the packages “MASS,” “tidyverse,” and “ggbeeswarm” were used in performing the ordinal logistic regression to generate the proxy-risk for introduction of CSFv in MT considering the model developed by ASF risk prediction for Kazakhstan (41). ArcGIS 10.5.1 (27) was used for spatial data processing and mapping data and results.

### Correlation Between Pathways

The correlation between the two pathways for the risk of introduction of CSF into MT was computed using a Spearman correlation test (R_s_) as

$$R_{s}=1-\frac{6\Sigma {\left(R_{i}-S_{i}\right)}^{2}}{n\left(n^{2}-1\right)}$$

(43)

where R_i_ is the rank for the value x_i_, which is the mean risk generated by risk assessment for the introduction of CSFv through movement of commercial pigs (Rpm), S_i_ is the rank for the value y_i_, which is the risk score generated by the assessment for the introduction of CSFv through movement of wild boars (Rbm), and n is the number of observations, i.e., the number of municipalities in MT (*n* = 141). The correlation was implemented in the RStudio Team (35) version 3.5.3 software using the statistics base-package “cor.test (x, y, method = ‘Spearman,’ exact = FALSE).”

Additionally, municipalities were categorized as low or high risk for each of the two pathways assessed. For the risk of introduction through movement of live pigs, we used 0.01 as the cutoff value because values lower than that would mean that, on average, one would expect one outbreak every 100 epidemics in the CSF-free zone, which is also relatively unexpected. For that reason, values <0.01 were assumed to represent negligible risk for this pathway. For the risk of introduction through wild boars, the median was used as a cutoff value to be able to divide the municipalities of MT as the 50% low and 50% high proxy-risk, allowing a conservative comparison. Both dichotomizations were subsequently combined to group municipalities into four categories, representing high risk to both, either (two groups), or none of the pathways.

## Results

**Figure 1:**
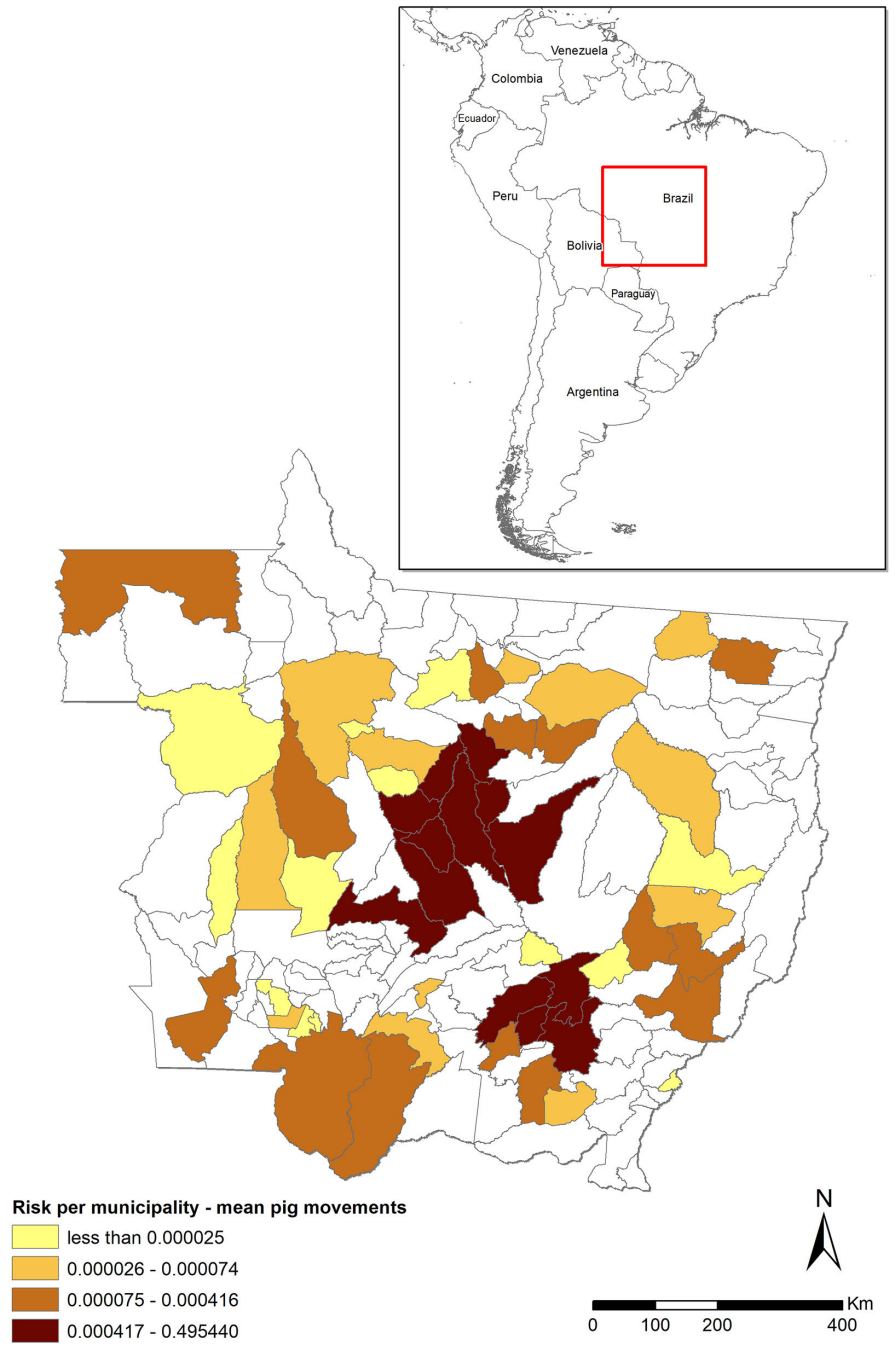
Risk of classical swine fever (CSF) introduction into Mato Grosso (MT) through movement of pigs (Rpm) stratified by municipality and assuming an undetected outbreak in states in the CSF-free zone that ship pigs to MT. The darker the shade, the higher the risk. Municipalities in white did not receive pigs from outside MT during the assessed 3-year period. The red square shows the localization of MT in Brazil/Latin America map.

**Figure 2:**
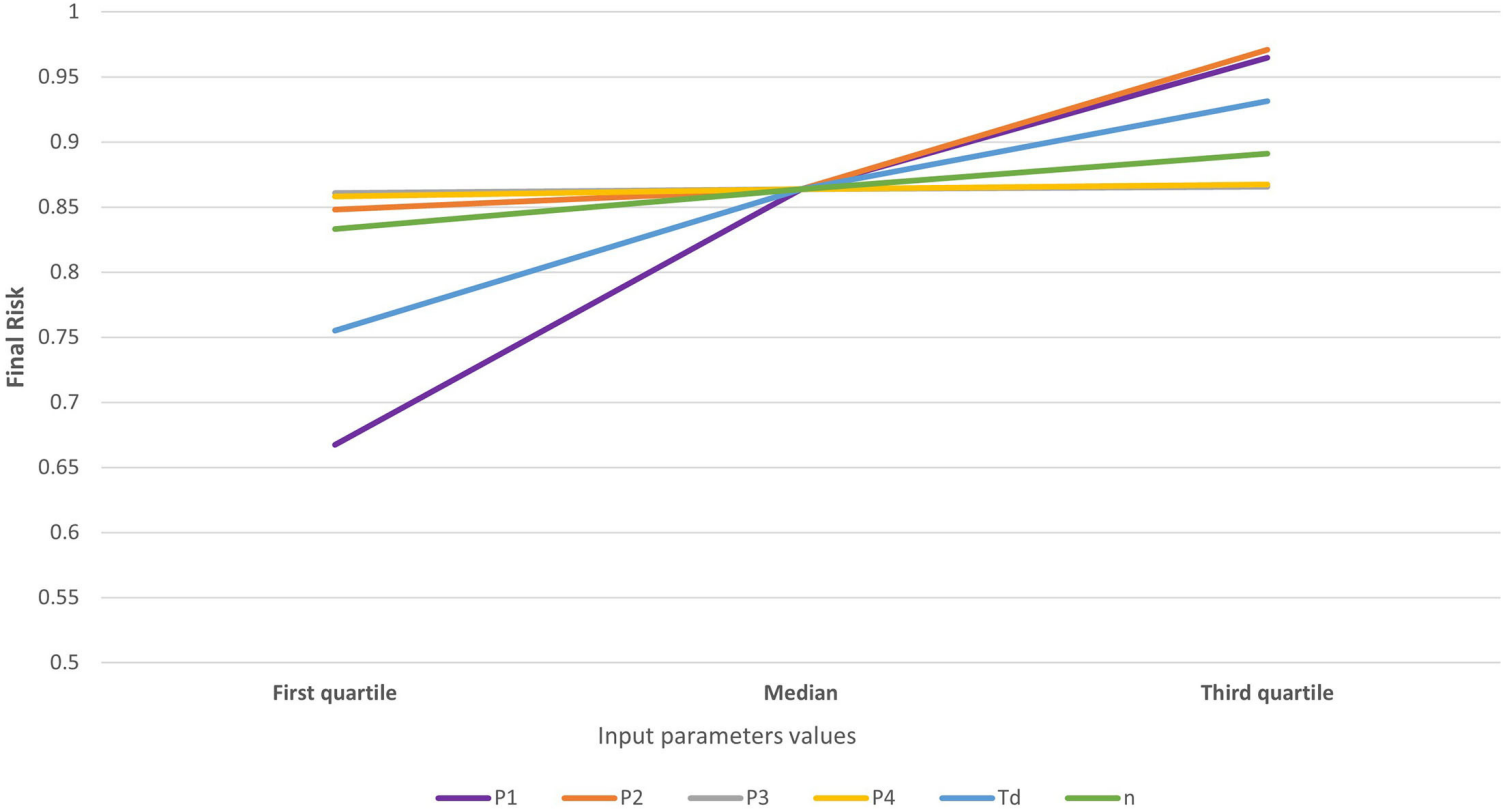
Sensitivity to variations in the parameters of a risk assessment model for the introduction of CSF into MT. Model parameters are the probability of importing an infected pig (P1—purple line), the probability that an infected pig survives the infection before the shipment to MT (P2—orange line), the probability that an infected pig survives the shipment to MT (P3—gray line), the probability that an infected imported pig established contact with a susceptible pig in a farm in MT (P4—yellow line), the time-to-detect the outbreak (Td—blue line), and the number of pigs shipped into MT (*n*—green line).

The risk associated with the legal movement of pigs (Rpm) was heavily concentrated, with five (3.5%) municipalities accounting for 96% of the total risk and much of the risk clustered in the central districts of MT (Figure 1). The risk was higher than the threshold (0.01) in only six municipalities, but it was relatively high (>0.1) in five of those six. In contrast, the risk was nil for most (*n* = 89, 63.1%) municipalities (Figure 1, districts in white) because they did not receive any pigs from outside MT from 2016 through 2018. The mean risk of introduction into MT [0.763−95% CI (0.21–1.0)] suggests that, in the scenario of a hypothetical outbreak in the CSF-free zone of Brazil and assuming that time-to-detection of the first outbreak would be similar to those observed in other epidemics, it is likely that MT would suffer an outbreak. The model was most sensitive to variations in the probability of importing an infected pig (P1) and the time-to-detection of the outbreak by the OVS at the origin (Td), followed by the probability of the pigs that survive the infection (P2) and the number of pigs shipped into MT (*n*), respectively (Figure 2).

**Figure 3:**
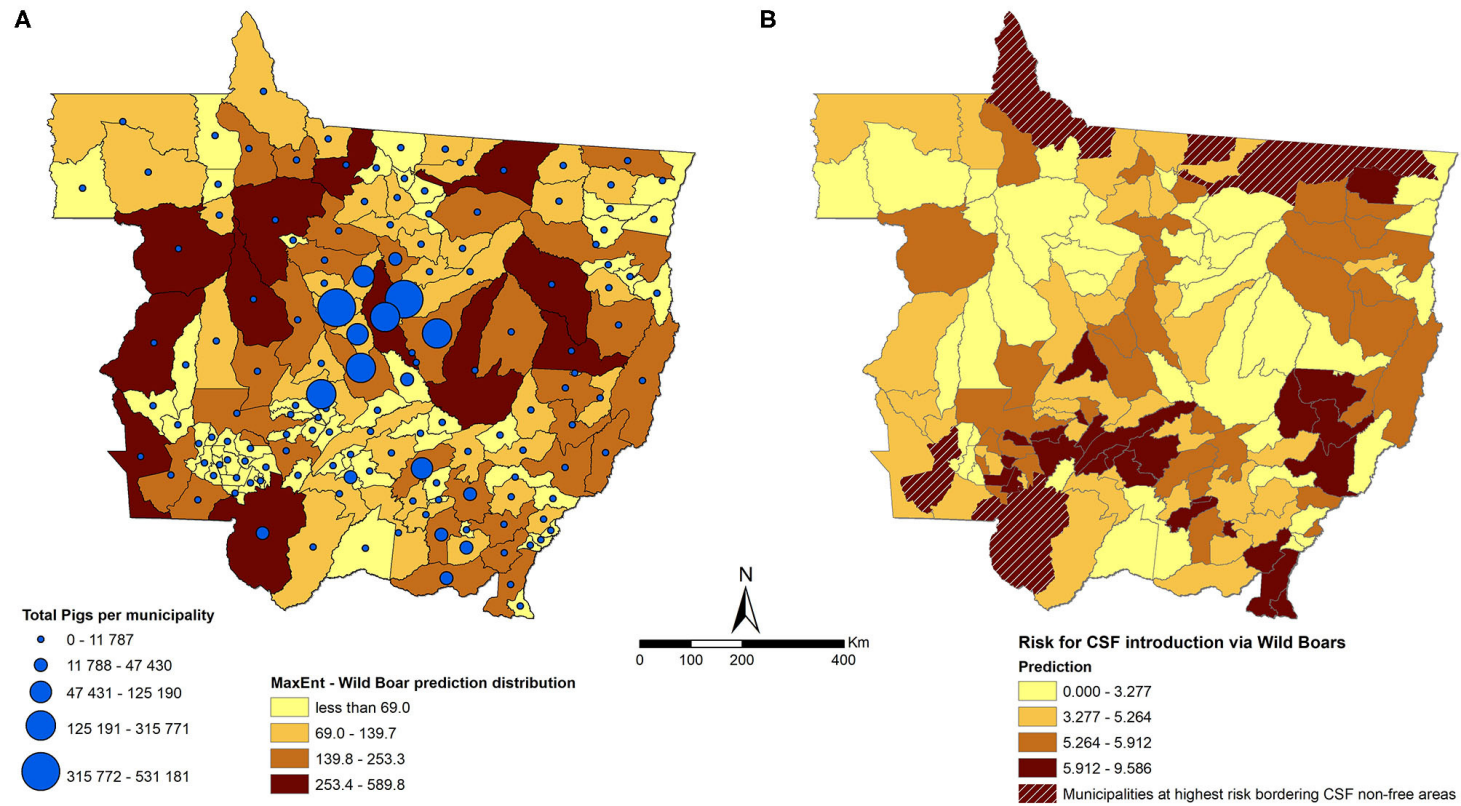
**(A)** Distribution of wild boars predicted by a maximum entropy model aggregated at the municipality level in MT (the darker the shade of the polygon, the higher the predicted value) and municipality-level number of pigs (the larger the size of the blue dot, the larger the number of pigs). **(B)** Results of the model for risk scores of the introduction of CSF into MT through wild boar movement (Rbm) (the darker the polygon, the higher the risk). The hatched areas are the municipalities at highest risk bordering CSF non-free areas.

The maximum entropy algorithm calculated the distribution of the wild boar population in MT using 1,048 observations of wild boar as a training data and 261 observations as a testing data from the total of 1,688 observations captured from active surveillance performed by OVS of MT from 2016 to 2018. Observations, 379, were excluded from the model due to issues with the geographic coordinates collected during the surveillance activity. The area under the curve (AUC) of the receiver operating characteristic (ROC) curve was 0.765 for testing, with 0.014 of standard deviation, which was considered an acceptable accuracy. Although wild boars were predicted to be distributed throughout the state (Figure 3A), most of the risk associated with CSFv introduction through free roaming of wild boars (as approximated by the value of the risk score Rbm) was concentrated in the northern and southern districts of MT (Figure 3B). Eight municipalities were estimated to be at the highest risk for introduction of CSF through wild boars, and these municipalities are bordering the non-CSF-free zone in the north of MT and Bolivia in the southwest (Figure 3B, hatched areas).

**Figure 4:**
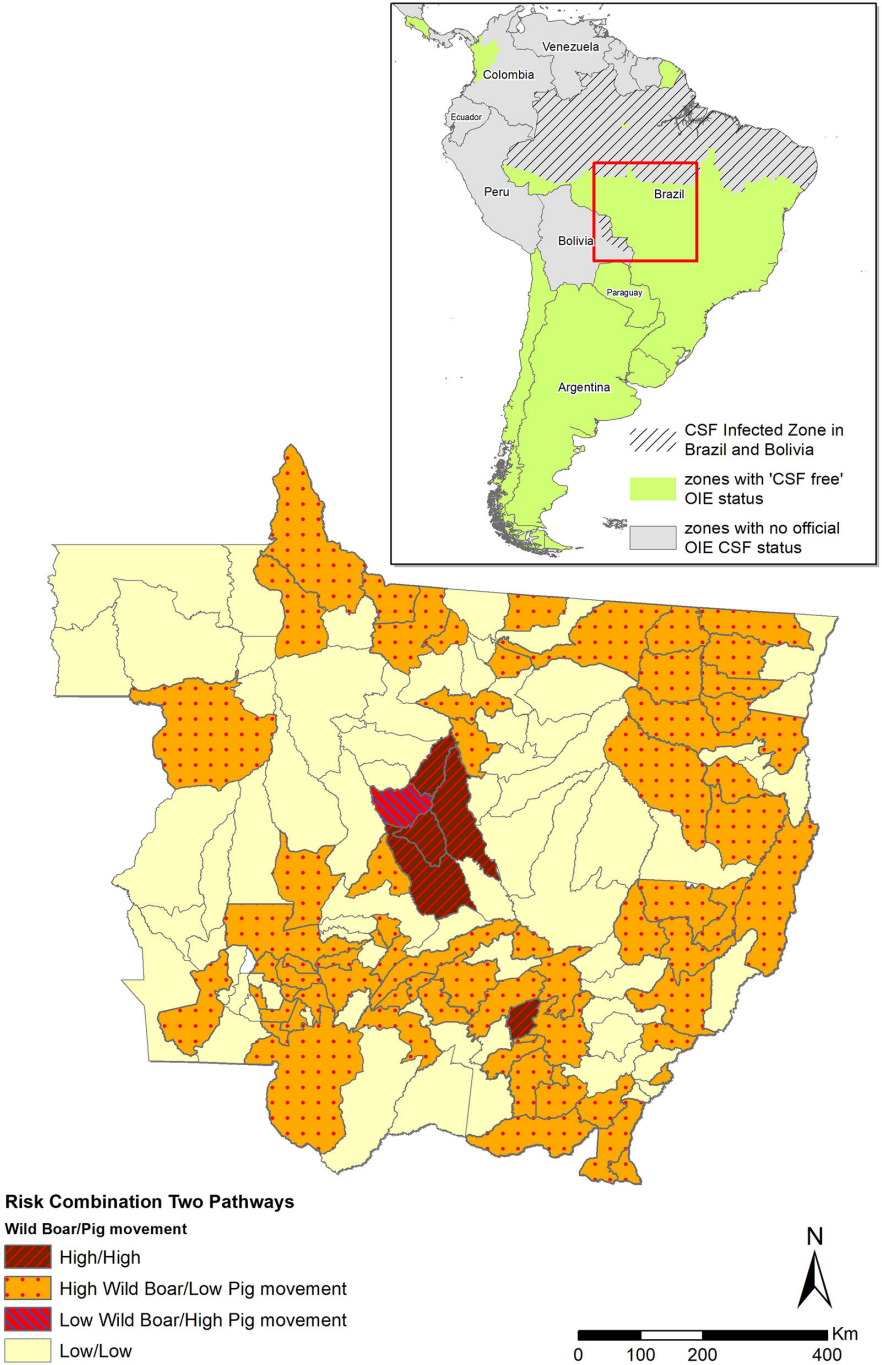
Risk for the introduction of CSF into MT through legal movement of pigs and through free roaming of wild boars, estimated using a combination of risk analysis models. Municipalities were categorized as high risk for both pathways (brown with red hatched area), high risk for wild boars and low risk for commercial pig movements (orange with red dots), low risk for wild boars and high risk for commercial pig movements (pink with blue hatched area), and low risk for both pathways (light yellow). The green area in the Latin America map (up right corner) shows the CSF-free area recognized by OIE. The hatched gray area shows the non-CSF-free zone.

The municipality-level risk for introduction of CSFv *via* movement of domestic pigs was poorly correlated (Spearman correlation coefficient, R_s_ = 0.11, *p*-value = 0.185) with the risk associated with free roaming of wild boars. Only five municipalities (four of them located in the central part of the state) were estimated at highest risk for introduction of CSF into MT through both pathways (Figure 4).

## Discussion

The work here characterized the risk associated with, arguably, two of the most important routes for introduction of CSF into MT, Brazil. We used these results to generate maps that depicted the spatial distribution of risk and identify municipalities that are most vulnerable to each of the assessed routes. Movement of live animals is one of the main routes for disease introduction into free areas (13). Other routes of introduction of CSF, such as legal or illegal contaminated pork products, contaminated trucks due to fecal contamination, genetic material from infected pigs such as semen, and human contact due to contamination clothing (10), were not specifically assessed here, and these results were restricted to the risk associated with movement of live pigs and wild boars. For the computation of the risk associated with wild boars, however, certain variables that may serve as proxy for unassessed routes, such as human and road density, were included in the model, which may have helped, in part, to account for that risk. If an outbreak occurs in the CSF-free zone, the economic impact will be devastating. In 2018, when some outbreaks in Brazil were detected in the CSF-non-free zone, the Confederation of Agriculture and Livestock of Brazil (CNA) estimated an impact of US$ 230–790 million if the infection reached the free zone of CSF in Brazil (40). For the risk assessment of introduction of CSFv in MT through movement of live pigs (Rpm), we considered a hypothetical scenario of an ongoing CSF outbreak in any other Brazilian State that is part of the CSF free-zone, with the intention to estimate the risk that MT would become infected when this occurs. The CSF-free zone is quite extensive, and the OVS of each state has its own surveillance system, which can impose variations for the time of detection outbreak, and this is a factor out of the control of MT. Ultimately, these results will help in evaluating the implementation of surveillance activities in MT and the prioritization of surveillance activities in relation to the route that imposes the highest risk for any given municipality.

The legislation that MT follows regarding CSF surveillance is dictated by the Brazilian Federal government, by which active serological surveillance is conducted only biannually in random backyard pig farms and in commercial farms only on months when the mortality rate exceeds the threshold for different ages or categories (44). However, the legislation does not consider the spatial heterogeneity of the risk imposed by alternative routes of entry. In states like MT, in which there are more than 40,000 registered pig farms, but only 550 of those are categorized as commercial farms, there is a need for specifying selective actions for municipalities, in alignment with the risk imposed by different routes, to complement the national regulation. For example, the correlation between the risk imposed by both routes was not significant (R_s_ = 0.11, *p*-value = 0.185), indicating that the districts estimated at highest risk for a given pathway were not at highest risk for the other route. However, because the risk for these two pathways was calculated using different methods, the raw values are not comparable. This finding is consistent with the need for enforcing different policy for districts regarding the design of surveillance and early detection strategies to prioritize practices associated with the routes that impose the highest risk to the municipality.

The Rpm, which was estimated assuming an undetected outbreak in the CSF-free zone of Brazil, was highly clustered in the central part of the State, where the largest pig farms in MT are located (Figure 1), with five municipalities concentrating 96% of the risk. A similar result was obtained in Spain, where risk was also concentrated in few provinces and in relation to those locations in which pig production is highly concentrated (11). Similar to a study conducted in Denmark, the risk associated with animal movements was relatively low, due to the small number of imported pigs (10). Another study had similar results, with overall low-risk probability for introduction of ASF/CSF into the US *via* legal import of pigs and pig products, and the highest values for the probability of introduction were concentrated in three US states traditionally associated with pig production (45). In MT, only a few municipalities account for most of the pigs moved from out of the state, and only those municipalities showed high mean risk probability. Thus, targeting a relatively low number of farms in those specific municipalities, for example, through enhanced passive surveillance protocols, would help to design surveillance strategies that account for most of the risk of introduction into MT through that route.

The sensitivity analysis showed that time-to-detection (Td) highly influences the risk. Because Td is expected to be the same for all municipalities, the variability of Td is not expected to affect the ranks estimated here. However, because the variability of Td may affect the likelihood of an outbreak, the sensitivity of results to the variability of the parameter contributes to highlighting the importance of coordination and collaboration between districts in Brazil, and the impact that early detection has in the mitigation of the impact of epidemics.

Although certain municipalities at the borders were found at highest risk for the introduction through wild boars (as approximated by the value of the risk score Rbm), we found that certain municipalities at the central region of MT were also at high risk, likely because of the combination of a number of factors, such as high density of humans and pigs and the presence of wild boars that would increase risk. The model used for the calculation of the risk in this pathway may outweigh the lack of a shared border with CSF-free areas, and the model did not require a shared border with CSF-free areas to have a negligible risk from this pathway. Certainly, some believe that the biggest challenge in maintaining a free or controlled area for CSF is for the OVS to be able to enforce control and eradication measures on subsistence pig farms (4). In those municipalities, surveillance efforts may be directed toward education and outreach actions involving small holders. Those outreach and education actions may be particularly challenging in MT, given that informal reports and anecdotal evidence suggest some backyard pig owners let sows commingle with wild boars to generate the strongest offspring, which increases the risk for CSF introduction. Wild boars play an important role in the environmental maintenance of CSF and its transmission to domestic pigs. In CSFv-infected regions in which there is a high density of wild boars, a situation of endemicity may be established (6). Targeted and strategic hunting may be considered as an action to reduce wild boar population and support the implementation of a surveillance program using samples obtained from hunted animals.

Epidemic models have been increasingly used to evaluate and inform disease surveillance and control policies. For that reason, risk assessments are important tools that should be routinely incorporated by OVSs to support the design of risk-based surveillance activities (46). Quantitative assessment of the risk for CSF introduction into a country or state may help the decision-making process to ultimately prevent and control disease introduction (2). Risk assessments combining different routes of introduction broaden the scope of results, enhancing the availability of information for guiding surveillance actions (47). Noteworthy, risk assessments are formulated considering a series of limitations and assumptions, and regular updates are required to evaluate if the conditions observed when formulating the models are still valid.

In conclusion, results here indicate that the risk for introduction of CSF into MT is spatially heterogeneous, suggesting that different approaches of targeted surveillance should be implemented in the state considering, at least, two primary objectives. On one hand, there is a need for increasing the number of OVS visits to commercial farms that receive animals from outside the state, inspecting and quarantining pigs as soon as they arrive at the farm, and considering the design of passive surveillance activities targeting the early detection of CSF-like signs in those particular farms and municipalities. On the other hand, for districts in which risk was mostly associated with wild boars, actions like sampling hunted wild boars and implementation of surveillance and educational and outreach programs in backyard farms should be prioritized (37). Results here will help MT to increase the efficiency of CSF surveillance, enhancing the federal rules for CSF surveillance actions, with the ultimate objective of preventing the introduction of the disease into the State.

## Data Availability Statement

The raw data supporting the conclusions of this article will be made available by the authors, without undue reservation.

## Author Contributions

DS led the data collection and analysis and wrote much of the paper. FK collaborated with the data analysis process and wrote some of the paper. AP supervised the design of the study, paper, and the data analysis process and wrote much of the paper. All authors contributed to the article and approved the submitted version.

## Conflict of Interest

The authors declare that the research was conducted in the absence of any commercial or financial relationships that could be construed as a potential conflict of interest.
